# Modulation of monocytes in septic patients: preserved phagocytic activity, increased ROS and NO generation, and decreased production of inflammatory cytokines

**DOI:** 10.1186/s40635-016-0078-1

**Published:** 2016-02-16

**Authors:** Sidnéia S. Santos, Amanda M. Carmo, Milena K. C. Brunialti, Flavia R. Machado, Luciano C. Azevedo, Murillo Assunção, Sílvia C. Trevelin, Fernando Q. Cunha, Reinaldo Salomao

**Affiliations:** Escola Paulista de Medicina, Hospital São Paulo, Universidade Federal de Sao Paulo, Sao Paulo, Brazil; Hospital Sírio Libanes, Universidade de Sao Paulo, Ribeirao Preto, Brazil; Hospital Israelita Albert Einstein, Universidade de Sao Paulo, Ribeirao Preto, Brazil; Faculdade de Medicina, Universidade de Sao Paulo, Ribeirao Preto, Brazil

**Keywords:** Monocytes, Reactive oxygen species, Nitric oxide, IL-6, TNF-α, Phagocytosis

## Abstract

**Background:**

The nature of the inflammatory response underscoring the pathophysiology of sepsis has been extensively studied. We hypothesized that different cell functions would be differentially regulated in a patient with sepsis. We evaluated the modulation of monocyte functions during sepsis by simultaneously assessing their phagocytic activity, the generation of reactive oxygen species (ROS) and nitric oxide (NO), and the production of inflammatory cytokines (IL-6 and TNF-α).

**Methods:**

Whole blood was obtained from patients with severe sepsis and septic shock both at admission (D0, *n* = 34) and after seven days of therapy (D7, *n* = 15); 19 healthy volunteers were included as a control group. The cells were stimulated with LPS, *Pseudomonas aeruginosa*, and *Staphylococcus aureus*. The ROS and NO levels were quantified in monocytes in whole blood by measuring the oxidation of 2,7-dichlorofluorescein diacetate and 4-amino-5-methylamino-2,7-difluorofluorescein diacetate, respectively. Intracellular IL-6 and TNF-α were detected using fluorochrome-conjugated specific antibodies. Monocyte functions were also evaluated in CD163+ and CD163− monocyte subsets.

**Results:**

The monocytes from septic patients presented with preserved phagocytosis, enhanced ROS and NO generation, and decreased production of inflammatory cytokines compared with the monocytes from healthy volunteers. TNF-α and IL-6 increased and ROS generation decreased in D7 compared with D0 samples. In general, CD163+ monocytes produced higher amounts of IL-6 and TNF-α and lower amounts of ROS and NO than did CD163− monocytes.

**Conclusions:**

We demonstrated that monocytes from septic patients, which are impaired to produce inflammatory cytokines, display potent phagocytic activity and increased ROS and NO generation.

## Background

Sepsis has been defined as a systemic inflammatory response (SIRS) triggered by an ongoing infection [1] and more recently considered as the host’s deleterious, non-resolving inflammatory response to infection that leads to organ dysfunction [2]. Understanding the nature of how the inflammatory response underscores the pathophysiology of sepsis would not only help clarify the mechanisms of the syndrome but would also lead to the identification of new therapeutic targets.

Currently, it is generally accepted that infection triggers both inflammatory and anti-inflammatory responses. Accordingly, two major mechanisms have been proposed for the injuries caused by sepsis: sustained activation of innate immunity leading to inflammation and injury [3] and a predominant initial hyperinflammatory phase followed by impaired immunity and an anti-inflammatory state [4].

One issue with this model is that the innate immune cells would be regulated in their global functions, and monocytes and neutrophils, for example, are thought to be suppressed in all of their activities in protracted septic patients [4]. In fact, most studies that evaluated blood cells from septic patients have demonstrated an impaired production of inflammatory cytokines after in vitro stimulation [4, 5], whereas neutrophils have been shown to have both up- and down-regulated functions [6]. Interestingly, we observed that peripheral mononuclear cells (PBMC) [7, 8] and monocytes [9] from septic patients, which were unable to produce inflammatory cytokines, showed an up-regulation of reactive oxygen species (ROS) generation [10], which was confirmed in another cohort of patients in whom the up-regulation of nitric oxide (NO) generation was also observed [11]. These findings indicate that both a hyperresponse and a hyporesponse can occur, depending on the functions and cells evaluated and, importantly, on the ongoing sepsis process [12, 13].

Reprogramming of monocyte functions was first proposed in an LPS-tolerance model where, depending on the preconditioning treatment, LPS induced selective priming effects on the production of TNF-α and NO in mouse peritoneal macrophages [14]. Subsequent studies demonstrated that LPS-tolerant cells do not produce inflammatory cytokines but present potent phagocytic activity and retain the ability to generate ROS [9, 15, 16]. The alternatively activated macrophages (AAM) also produce reduced levels of inflammatory cytokines and exhibited regulatory or repair activity [17]. These cells exhibited an increased expression of CD206 (mannose receptor) and CD163 (hemoglobin-haptoglobin receptor) [18] receptors, considered to be typical markers of AAMs.

There is a great interest to study CD163 in sepsis. As a receptor expressed on AAM, it might be a surrogate marker of monocytes and macrophages modulation during sepsis. CD163 also functions as an innate sensor for bacteria [19], and activation of cell surface Toll-like receptors induces shedding of the receptor, as an acute response to extracellular pathogens [20]. Finally, as a scavenger of Hb, CD163 contributes to the anti-inflammatory response. In clinical settings, increased detection of membrane-bound and soluble CD163 has been reported in septic patients [21, 22].

We hypothesized that different cell functions would be differentially regulated in a patient with sepsis. Thus, we evaluated monocyte modulation during sepsis by simultaneously assessing their phagocytic activity, the generation of ROS and NO, and the production of inflammatory cytokines (IL-6 and TNF-α). Furthermore, we determined if the modulation of monocytes’ function during sepsis is associated with the phenotype of cells expressing CD163.

## Methods

### Patients and healthy volunteers

Patients admitted to the intensive care units of the Sao Paulo, Albert Einstein, and Sirio-Libanes Hospitals with a clinical diagnosis of sepsis according to the ACCP/SCCM consensus conference [1], from April 2014 to June 2015, were enrolled in the study. The protocol was approved by the ethics committees of the participating hospitals.

Blood samples were obtained from 34 septic patients at admission (D0), and 15 of the patients had a second sample collected after 7 days (D7) of therapy. Samples were also collected from 19 healthy volunteers who were matched according to age and gender.

### LPS, gram-negative, and gram-positive bacteria

LPS from *Salmonella abortus equi* was a generous gift from C. Galanos (Max-Planck Institute of Immunobiology, Germany). *Pseudomonas aeruginosa* (ATCC27853) and *S. aureus* (ATCC 25923) were purchased from Oxoid Limited, Basingstoke, Hampshire, UK.

### Induction and detection of the production of ROS and NO in monocytes in whole blood

ROS and NO were measured constitutively and after stimulation with LPS and heat-killed *S. aureus*, and *P. aeruginosa* for 30 min. Based on the dose-response curves, 100 ng/mL LPS and 2.4 × 10^8^ colonies/mL *S. aureus* were used for induction of ROS and NO. The concentration of *P. aeruginosa* was 2.4 × 10^7^ colonies/mL for ROS and 2.4 × 10^8^ colonies/mL for NO. The ROS and NO levels were quantified in monocytes in whole blood by measuring the oxidation of 2,7-dichlorofluorescein diacetate (DCFH-DA; Sigma, St. Louis, MO) and 4-amino-5-methylamino-2,7-difluorofluorescein diacetate (DAF-FMDA; Invitrogen, Carlsbad, CA), respectively, as previously described [11, 23]. Briefly, the tubes from each sample were incubated in the presence of 0.06 mM DCFH-DA or 0.01 mM DAF-FMDA in a 37 °C shaking water bath for 30 min. After incubation, 2 mL of 3 mM EDTA (Sigma) or phosphate-buffered saline (PBS) was added to each tube for ROS and NO determination, respectively, and the mixture was then centrifuged (800*g* for 5 min at 4 °C). Erythrocytes were lysed in hypotonic saline, and the pellets were incubated with 6 μL of CD14-PerCP clone MΦP9 (BD Bioscience, San Jose, CA, USA) and anti-CD163-PE clone GHI/61 (BD Bioscience) at room temperature for 15 min in the dark. Then, 2 ml of PBS was added to each tube, and the mixture was centrifuged (800*g* for 5 min at 4 °C). The supernatants were discarded, and the pellets were resuspended in 300 μL of PBS for flow cytometric analysis.

### Intracellular detection of cytokines in monocytes in whole blood

Whole blood was diluted 1:2 in RPMI and incubated with LPS and heat-killed bacteria (LPS: 100 ng/mL, *P. aeruginosa* and S*. aureus*: 2.4 × 10^8^/mL), or without stimulus in 5-mL propylene tubes at 37 °C in the presence of 5 % CO_2_. After 30 min, 5 μL (1 mg/mL) of Brefeldin A (Sigma, Saint Louis, MO, USA) was added to the samples, and they were incubated for an additional 4 h. After washing, the red blood cells were ruptured with 2 mL lysis solution (FACS lysing solution, BD Bioscience). After washing with 2 mL PBS, the samples were incubated with the fluorochrome-conjugated monoclonal antibodies CD14-PerCP clone MΦP9 (BD Bioscience) and anti-CD163-PE clone GHI/61 (BD Bioscience) for surface staining for 15 min in the dark at room temperature. The samples were washed in 2 mL PBS, centrifuged, and fixed with 500 μL fixation buffer (PBS 4 % paraformaldehyde) for 30 min in the dark at 4 °C. After centrifugation, 50 μL permeabilization buffer (PBS 1 % FCS; 0.1 % saponin), anti-IL-6-APC clone MQ2-13A5 (BD Bioscience), and anti-TNF-PE-Cy7 clone Mab11 (BD Bioscience) were added to the tubes. The tubes were incubated for 30 min in the dark on ice. Then, the samples were washed with 2 mL permeabilization buffer, and the cells were suspended in Macs buffer for flow cytometric analysis [15].

### Phagocytosis of monocytes in whole blood

Phagocytosis of monocytes was measured using *Escherichia coli* conjugated to FITC (Phagotest™, Glycotope Biotechnology, Heidelberg, Germany), accordingly to the manufacturer instructions.

### Flow cytometry

Detection of phagocytosis and the production of ROS, NO, IL-6, and TNF-α by monocytes in whole blood was performed by multiparameter flow cytometry (LSRFORTESSA (BD Bioscience)). Events acquisition was performed using FACSDiva software (BD Bioscience). For detection of the production of ROS, NO, IL-6, and TNF-α by monocytes, 5000 events were acquired using forward- and side-scatter parameters combined with CD14-positive cells. For the detection of phagocytosis, 15,000 events were acquired using forward- and side-scatter parameters to determine the monocyte population. All events were acquired and stored, and the analysis was performed using FlowJo (Tree Star INC. Ashland, OR, USA).

#### Detection of the production of ROS, NO, IL-6, and TNF-α

Monocyte analysis was performed by assessing individual cells (singlets) combined with side-scatter parameters versus CD14 positiveness. Monocytes were further characterized as CD163+ or CD163− cells. The quadrant for CD163+ cells was established based on isotype control.

**Fig. 1:**
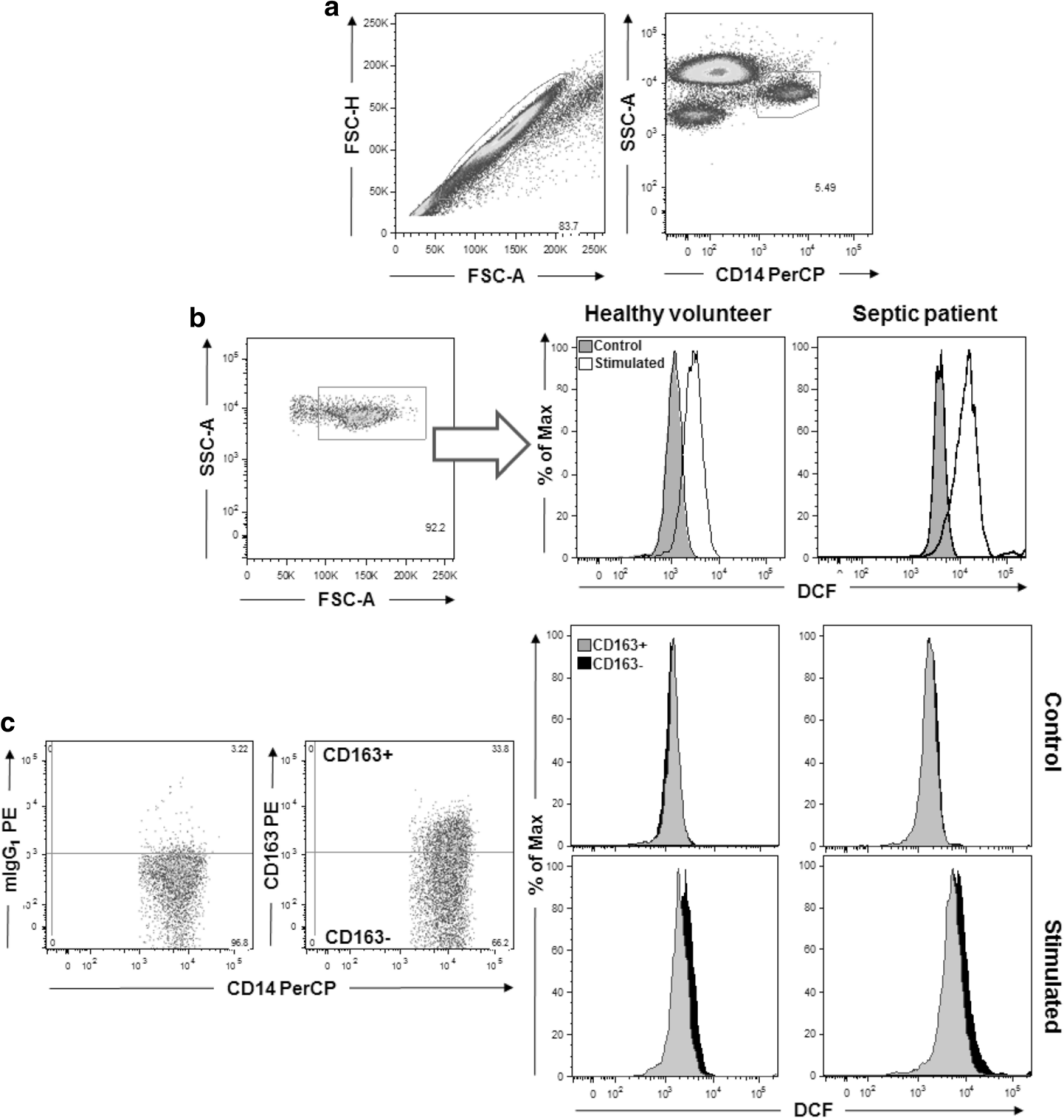
Analysis charts to detect the production of ROS and NO in monocytes and subpopulations. Individual cells (singlets) were selected, and monocytes were characterized by light scatter and side positivity for CD14 (**a**). Another side versus forward light scatter plot was used to exclude smaller cells (**b**). CD163+ and CD163− subsets were established based on isotype control (**c**). ROS generation was measured by GMFI of DCFH represented in histogram graphics for the CD14+ monocytes and the monocyte subsets CD14+ CD163+ and CD14+ CD163−. The graphics are representative of an experiment for ROS detection in healthy volunteers and septic patients in unstimulated cells and after stimulation with *P. aeruginosa*

The productions of ROS and NO were analyzed in monocytes and in the subsets of CD163+ and CD163− monocytes in histogram charts. They were quantified by the geometric mean fluorescence intensity (MGIF) associated with the detection of DCFH and DAF, respectively (Fig. 1). Under the experimental conditions for oxidative metabolism measurement, the expression of CD163 on monocytes was 50.5 ± 17.7 % (mean ± SD) in septic patients and 21.3 ± 20.2 % in healthy volunteers.

**Fig. 2:**
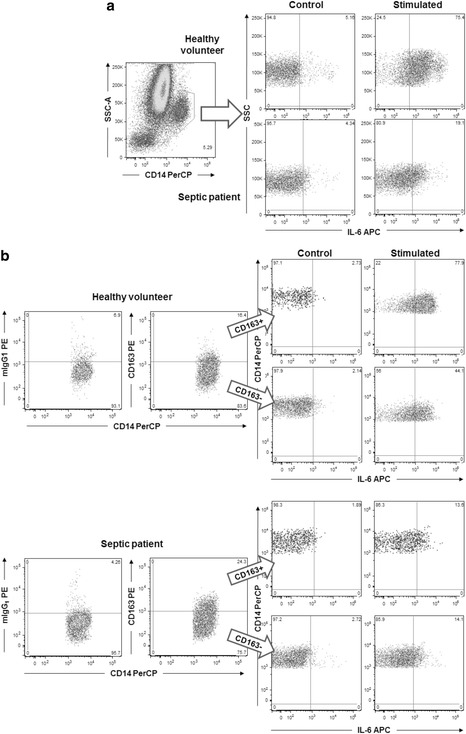
Strategy to detect intracellular cytokine (IL-6) in monocytes and CD163+ and CD163− subsets. **a** Individual cells (singlets) were selected, and monocytes were characterized by light scatter and side positivity for CD14. *Dot plots* were drawn with IL-6 versus the side scatter parameter. The quadrants for IL-6 positivity were established based on unstimulated cells (control), and the percentages of positive stained cells were determined after stimulation. **b** CD163+ and CD163− subsets of monocytes were established based on isotype control, and the percentages of cells positively stained for IL-6 were measured after stimulation. The graphics are representative of an experiment for IL-6 detection in a healthy volunteer and in a septic patient in unstimulated cells and after stimulation with *P. aeruginosa*

Intracellular cytokine levels were analyzed both in monocytes and in the subsets of CD163+ and CD163− monocytes based on the quadrants established in the sample without stimulation and are expressed as the percentage of cytokine-producing monocytes (Fig. 2). Under the experimental conditions for intracellular cytokines detection, the expression of CD163 on monocytes was 41.6 ± 4.4 % (mean ± SD) in septic patients and 30.9 ± 18.9 % in healthy volunteers.

### Co-location of gp91phox and p47phox by immunofluorescence

PBMCs were obtained using the Ficoll density gradient method (Ficoll-Paque PLUS; GE Healthcare Bio-Sciences AB, Uppsala, Sweden) and stored in liquid nitrogen until use. After defrosting, the cells were spun on glass slides. The cells were incubated overnight with the primary antibodies goat anti-Nox2 (1:200) and rabbit anti-p47 (1:100) and then incubated with red fluorescent Alexa Fluor 594 (donkey anti-goat; 1:400), and/or green fluorescent Alexa Fluor 488 (donkey anti-rabbit; 1:200). Nuclear material was stained with 4, 6-diamidino-2-phenylindole (DAPI; Sigma-Aldrich, USA). Images of stained cells were captured using a confocal microscope SP5 (Leica, USA). The images were analyzed in the program ImageJ (National Institutes of Health, Bethesda, Maryland, USA) using the plugincolocalizationanalysis/colocalizationhighlighter (co-localized points—8 bit). That tool generated a new image that presented the points of co-localization of p47phox and gp91phox. Those points of co-localization were quantified from the average fluorescence intensity corresponding to two to four cells/randomly selected field.

#### Statistical analysis

The results were analyzed using SPSS (Statistical Package for Social Sciences v 19.0) (IBM, Armonk, NY, USA). The Shapiro-Wilk test was applied to determine the normality of the results. Comparisons between healthy volunteers and patients were performed using the Mann-Whitney *U* test, and comparisons between patient samples (D7 vs. D0) were performed using the Wilcoxon signed-rank test. Group comparisons were performed by using the Kruskal-Wallis test. The variables that showed differences among the three groups were compared group to group by the Mann-Whitney test.

The interactions of CD163 with ROS, NO, IL-6, and TNF levels were analyzed by two-way repeated measures analysis of variance (ANOVA) with the Bonferroni posttest. *P* values ≤0.05 were considered significant.

## Results

### Patient demographic and clinical data

Thirty-four patients with severe sepsis and septic shock and 19 healthy volunteers matched for gender and age were enrolled in the study. The clinical and demographic data of the patients is shown in Table 1. The mean age of the healthy volunteers was 59.9 years, ranging from 30 to 88 years, and 52.6 % were male.

**Table 1:** Demographic data and outcomes from septic patients included in the study

Cohort of septic patients (*n* = 34)	
Age [mean (SD)]	62.4 (19)
Gender [*N* (%)]	
Male	18 (52.9)
Female	16 (47.1)
Stages of sepsis [*N* (%)]	
Severe sepsis	19 (55.9)
Septic shock	15 (44.1)
SOFA score (D0)	5.5 (1–16)
In hospital mortality [*N* (%)]	
Survivors	30 (88.2)
Non-survivors	4 (11.8)
Outcome accordingly to stage at enrollment [*N* (%)]	
Severe sepsis	
Survivors	19 (100)
Non-survivors	0 (0)
Septic shock	
Survivors	11 (73.4)
Non-survivors	4 (26.6)
Sources of infection [*N* (%)]	
Respiratory tract	16 (47.1)
Abdomen	7 (20.6)
Urinary tract	8 (23.5)
Others	3 (8.8)

*SOFA* sequential organ failure assessment

### Phagocytosis, ROS and NO production, and intracellular detection of cytokines in monocytes in whole blood

No differences were found in monocyte phagocytosis of opsonized *E. coli* between healthy volunteers (median, GMFI, 15.499; range 8.722–24.879) and septic patients at D0 (median, GMFI, 19.707; range 5.207–35.075) (*P* = 0.178). Similarly, no differences were found when patients were classified as having severe sepsis or septic shock: severe sepsis (median, GMFI, 23.733; range 6.118–35.075) and septic shock (median, GMFI, 17.116; range 5.207–32.877) (*P* = 0.112).

ROS and NO generation were higher in septic patients than in healthy volunteers in all conditions tested (Table 2). In contrast, the percentages of monocytes producing TNF-α and IL-6 were lower in septic patients than in healthy volunteers following LPS, *P. aeruginosa*, and *S. aureus* stimulation (Table 2).

**Table 2:** Production of ROS, NO, IL-6, and TNF-α by monocytes of septic patients and healthy volunteers

	Septic patients			Healthy volunteers			
	Median	Percentiles 25–75		Median	Percentiles 25–75		
ROS (GMFI)							*P* ^a^
Control	2459	1735	4546	1005	761	1487	<0.001
LPS	3387	2049	6205	1177	829	1503	<0.001
*P. aeruginosa*	3972	2257	6514	1372	928	1560	<0.001
*S. aureus*	9296	5573	18507	2479	1749	3275	<0.001
NO (GMFI)							
Control	479	362	621	209	176	305	<0.001
LPS	572	448	792	284	243	389	<0.001
*P. aeruginosa*	917	631	1493	517	422	667	<0.001
*S. aureus*	840	617	1103	372	309	663	<0.001
IL-6 (%)							
LPS	10.9	4.9	41.2	70.8	60.2	77.5	<0.001
*P. aeruginosa*	24.1	4.9	51.8	70.4	54.1	78.1	<0.001
*S. aureus*	9 · 6	4.1	25.1	24.8	18.9	41.9	0.002
TNF-α (%)							
LPS	18.6	6.5	36.2	66.7	47.8	72.1	<0.001
*P. aeruginosa*	33.4	17.5	56.8	70.7	61.4	86.8	<0.001
*S. aureus*	19.6	9.3	42.4	33.6	26.5	43.9	0.023

Values for ROS and NO are shown as geometric mean fluorescence intensities (GMFIs) of DCFH and DAF, respectively. Cytokine values are shown as percentages of cells producing IL-6 and TNF-α

^a^Mann-Whitney *U* test

**Fig. 3:**
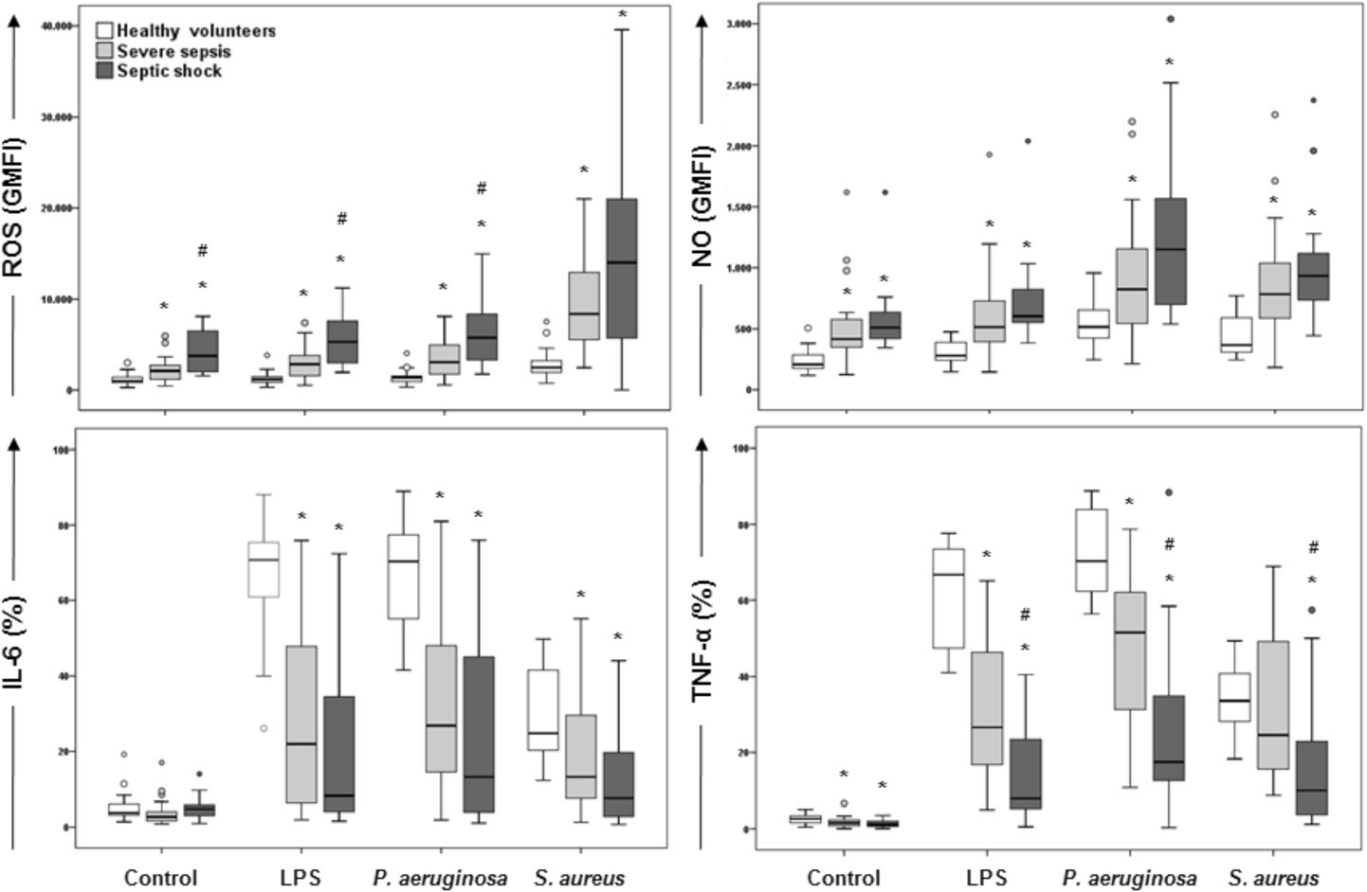
Detection of ROS, NO, IL-6, and TNF-α in monocytes of healthy volunteers and patients with severe sepsis and septic shock. Whole blood was obtained from 19 healthy volunteers (*blank boxes*), 19 patients with severe sepsis (boxes in *light gray*), and 15 patients with septic shock (boxes in *dark gray*). Monocyte analysis was performed by assessing individual cells (singlets) combined with the side-scatter parameter versus CD14 positiveness. The values for ROS and NO are shown as geometric mean fluorescence intensities (GMFI) of DCFH and DAF, respectively. Cytokine values are shown as percentages of cells producing IL-6 and TNF-α. *P* < 0.001 for all stimuli compared between groups (Kruskal-Wallis). *P < 0.05 compared to healthy volunteers, ^#^ *P* < 0.05 compared to severe sepsis patients (Mann-Whitney)

ROS and NO generation differed when healthy volunteers, severe sepsis patients, and septic shock patients were compared in all conditions tested (*P* < 0.001, Kruskal-Wallis) (Fig. 3a, b). The pairwise comparison (Mann-Whitney) showed that patients with severe sepsis and septic shock had higher ROS and NO generation than did healthy volunteers in all conditions tested (Fig. 3a, b). ROS generation was higher in septic shock patients than in sepsis patients in unstimulated cells and after LPS, and *P. aeruginosa* stimulation (Fig. 3a), whereas no differences were found in NO production between patients with severe sepsis and septic shock (Fig. 3b).

The percentages of monocytes producing IL-6 was lower in severe sepsis and septic shock patients than in healthy volunteers following LPS, *P. aeruginosa*, and *S. aureus* stimulation (*P* < 0.05, Kruskal-Wallis); no differences were found between patients with severe sepsis and septic shock (Fig. 3c).

Similarly, TNF-α production differed among healthy volunteers, severe sepsis patients and septic shock patients in all conditions tested (*P* < 0.05, Kruskal-Wallis). In this case, differences were found in the septic group, with lower detection in patients with septic shock than in those with severe sepsis for all stimuli tested (Fig. 3d).

ROS generation was positively correlated with SOFA score in the control condition (*R* = 0.371, *P* = 0.034) and after LPS (*R* = 0.414, *P* = 0.017) and *P. aeruginosa* (*R* = 0.409, *P* = 0.018) stimulation, but not with *S. aureus* (*R* = 0.109, *P* = 0.545). No correlations were found between the organ dysfunction score and any other cell functions evaluated in any of the conditions tested (Pearson correlation test).

### Interaction between monocyte functions and cell surface expression of CD163

**Fig. 4:**
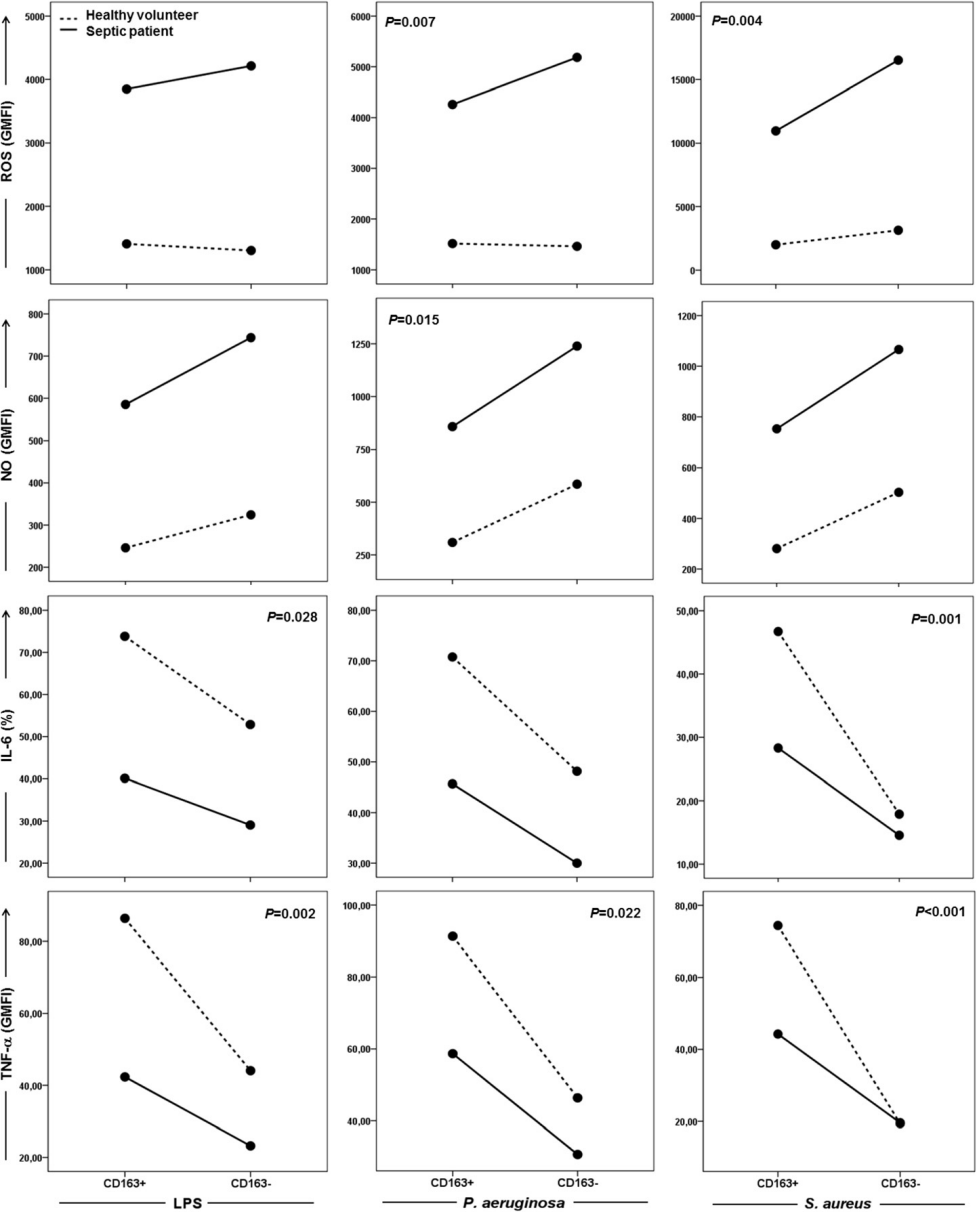
Interaction of CD163 expression on monocytes with the production of ROS, NO, IL-6, and TNF-α. Whole blood of 34 septic patients (D0) (*solid line*) and 19 healthy volunteers (*dot line*) were analyzed after stimulation with LPS, *P. aeruginosa*, and *S. aureus*. Values represent the means for each variable in CD163+ and CD163− monocytes. Monocyte analysis was performed by assessing individual cells (singlets) combined with side-scatter parameters versus CD14 positiveness. Monocytes were further characterized as CD163+ and CD163− cells. The productions of ROS and NO were analyzed in histogram charts and quantified as the geometric mean fluorescence intensities (GMFIs) associated with the detection of DCFH and DAF, respectively. Intracellular cytokine level was based on quadrants established in the sample without stimulation and are expressed as the percentage of cytokine-producing monocytes. *P values denote the interaction between CD163 expression and the groups of healthy volunteers and septic patients for each parameter and condition evaluated (ANOVA)

We assessed whether the differences in the modulation of the generation of ROS, NO IL-6, and TNF-α observed between septic patients and healthy volunteers were influenced by CD163 expression on the surfaces of monocytes. CD163 expression was found to be associated with IL-6 production after stimulation with LPS and *S. aureus* and with TNF-α production after stimulation with LPS, *P. aeruginosa*, and *S. aureus* (Fig. 4). In all conditions, CD163+ monocytes produced higher amounts of cytokines than CD163− monocytes in both septic patients and healthy subjects (*P* < 0.05). The levels of TNF-α and IL-6 were higher in healthy volunteers than in septic patients in both CD163+ and CD163− monocytes (*P* < 0.05) in all tested conditions, except for CD163− monocytes after stimulation with *S. aureus* (*P* = 0.958). An interaction was also found between CD163 expression and ROS generation after stimulation with *S. aureus* and *P. aeruginosa*. The difference in ROS generation between CD163− and CD163+ cells was only observed in septic patients, with CD163− cells producing higher amounts of ROS (*P* < 0.001). ROS generation was higher in septic patients than in healthy volunteers following S*. aureus* and *P. aeruginosa* stimulation in both CD163− and CD163 + monocytes (*P* < 0.001). Finally, an interaction between CD163 expression and NO generation was found after LPS stimulation, with the highest values observed in CD163− cells (Fig. 4).

### Dynamics of monocyte functions in patient follow-up samples

There was no difference in the phagocytic activity of monocytes from septic patients at admission (D0) (Median, GMFI, 19.473; range 5.207−35.075) and after 7 days of follow-up (Median, GMFI, 18.887; range 6.023−31.803) (*P* = 0.875).

**Fig. 5:**
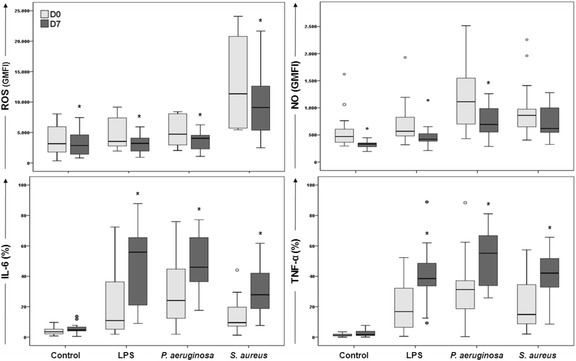
Detection of ROS, NO, IL-6, and TNF-α in monocytes of patients in admission and follow-up samples. Samples of 15 septic patients were assessed at D0 and D7 under different stimuli. Monocyte analysis was performed by assessing individual cells (singlets) combined with the side-scatter parameter versus CD14 positiveness. Data are shown as the geometric mean fluorescence intensities (GMFIs) of DCFH and DAF for ROS and NO, respectively, and as the percentages of cells producing IL-6 and TNF-α. *P < 0.05 compared to D0 (Wilcoxon)

ROS generation was lower at D7 than at D0 in all conditions tested (Fig. 5). A similar trend was seen for NO generation, but a significant change was only observed after *P. aeruginosa* stimulation (Fig. 5). In contrast, increased levels of IL-6 and TNF-α were found at D7 compared with those observed at D0 following LPS, *P. aeruginosa*, and *S. aureus* stimulation (Fig. 5).

Analysis of groups, including healthy volunteers and patients at D0 and D7, showed that differences between D7 and healthy volunteers were no longer significant for NO after *P. aeruginosa* and IL-6 and TNF-α after *S. aureus* stimulation.

### Co-location of gp91phox and p47phox by immunofluorescence

**Fig. 6:**
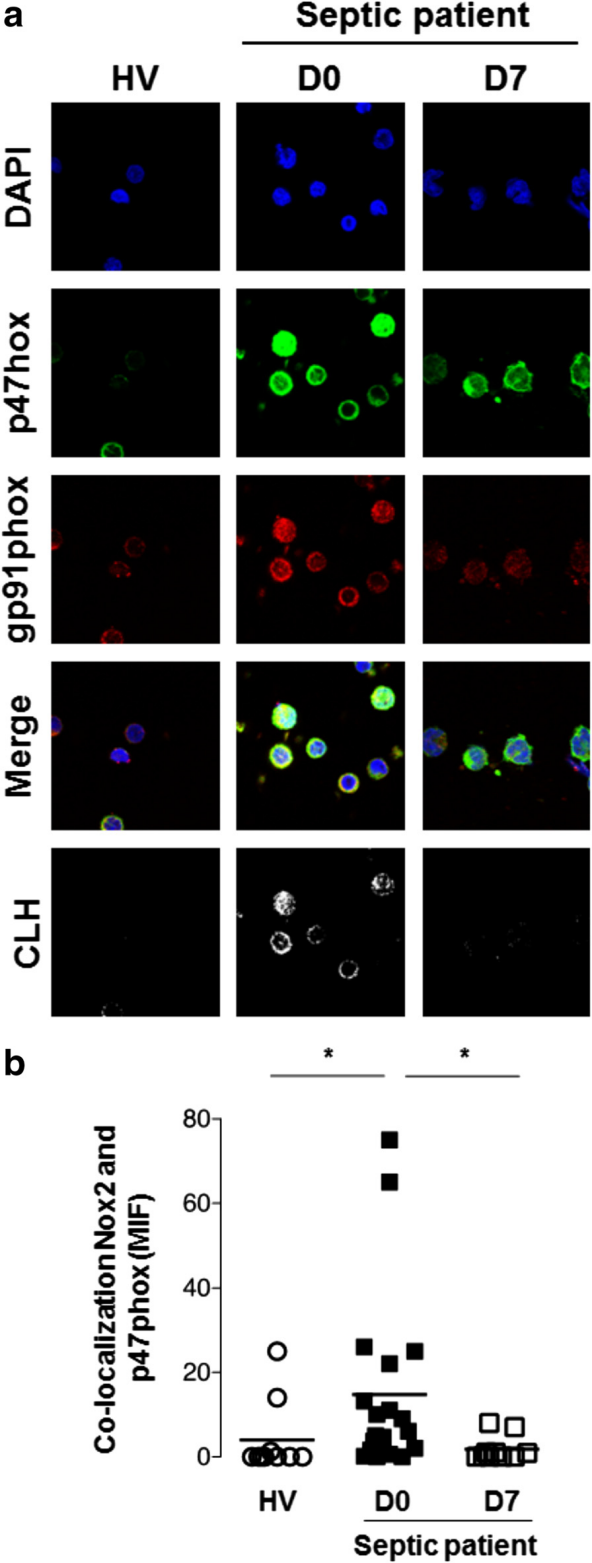
Co-localization of gp91phox (*red*) and p47phox (*green*) by immunofluorescence. PBMCs were obtained from septic patients at admission (D0; *n* = 20) and after 7 days of follow-up (D7; *n* = 10) and from healthy subjects (*n* = 10). **a.** The cell nuclei were stained with 4,6-diamidino-2-phenylindole (DAPI, *blue*) and gp91phox and p47phox using specific antibodies. The images were obtained using a confocal microscope (objective ×60; ×5 amplification). **b.** The graph represents the mean fluorescence intensity (MFI) obtained by co-localization analysis (colocalizationhighlighter) using ImageJ software (National Institutes of Health, Bethesda, MD, USA). *P < 0.05

At D0, monocytes from septic patients presented with higher Nox2 activation, as assessed by co-location of gp91phox and p47phox, than did monocytes from healthy volunteers. A significant decrease in Nox2 activation was observed after 7 days of follow-up (D7) (*P* < 0.05) (Fig. 6).

## Discussion

Our results show that monocytes from septic patients are modulated during the ongoing infection process, with preserved phagocytosis, increased ROS and NO generation, and decreased production of inflammatory cytokines. These results are consistent with our previously reported findings obtained in monocytes from septic patients [7, 9–11] and further support the concept of “reprogramming,” or modulation of cell functions rather than hyporesponsiveness during sepsis [12, 24].

These results also indicate the similarities between monocyte modulation in LPS-tolerance models and monocyte modulation in sepsis [12, 15, 16]. Multiple mechanisms have been shown to be involved in tolerance to LPS. Foster et al. reported the epigenetic mechanisms driving the modulation of LPS-response in LPS-tolerant cells. They found two groups of differentially regulated genes: the “tolerizeable” (T) and the “nontolerizeable” (NT) genes. The pro-inflammatory cytokine genes were found to be down-regulated (T), whereas antimicrobial genes were found to be up-regulated (NT), thus supporting their hypothesis that TLR-induced gene expression with different biological functions is distinctly regulated [25]. These findings were extended to human monocytes by Del Fresno and coworkers, who found down-regulation of pro-inflammatory cytokines and antigen presentation genes and up-regulation of anti-inflammatory factors, such as IRAK-M, and antimicrobial effectors [16]. In our own study, which focused on the TLR pathway, we observed down-regulation of TNF-α, IL-12, and CCL2 and up-regulation of IL-10 and colony stimulating factors (CSF2 and CSF3) in tolerant cells [26].

Down-regulation of inflammatory cytokines, measured at intracellular level in our study, has been consistently reported in the literature upon the in vitro stimulation of monocytes from septic patients [8, 27, 28]. Modulation of the monocyte response during sepsis occurred despite preservation of LPS binding to monocytes and of TLR2 and TLR4 expression on the monocyte cell surface [7, 9, 13]. The regulation of IL-10 production is more controversial. In this study, we found no differences in intracellular levels of IL-10 in monocytes in a subset of patients (*N* = 12) and healthy volunteers (*N* = 12) (data not shown); this finding is consistent with our previous results in whole blood supernatants [8].

Monocytes in whole blood presented increases in ROS and NO generation in vitro after stimulation with LPS, and Gram-negative and Gram-positive clinically significant bacteria, *P. aeruginosa*, and *Staphylococcus aureus*, respectively. This finding is consistent with our previously reported results in two other series of septic patients [10, 11]. To further link ROS generation to phagocytosis, we evaluated the co-localization of p47^phox^ and NOX-2 (gp91^phox^) in monocytes of septic patients. Co-localization was found in septic patients, mainly in the admission samples, and not in healthy volunteers, indicating that increased NADPH-oxidase activity is a source of ROS in septic patients. In addition to the role of ROS in antimicrobial defense, ROS is associated with cell and organ toxicity in sepsis. Consistent with previous findings [10], we found that ROS generation correlated with the SOFA score in most conditions.

In the follow-up samples, decreased production of ROS and increased production of inflammatory cytokines were observed under all stimuli compared to the admission samples, which indicated a trend toward the restoration of homeostasis. Interestingly, under *S.aureus*, stimulation levels of IL-6 and TNF-α in patients’ follow-up samples did not differ from healthy volunteers.

In further support of modulation rather than hyporesponse in monocytes during sepsis, we found that the phagocytic activity of monocytes was preserved during the ongoing infection process, even in patients with septic shock. This finding is in agreement with previous studies of LPS-induced tolerance in vitro [15, 16].

In addition to the above described similarities with LPS-tolerant monocytes, the pattern of activities of monocytes from septic patients in this study resembles that described for macrophages under the effects of pro-resolving mediators, which present enhanced phagocytic activity without evoking pro-inflammatory responses [29].

We evaluated whether the differences in the modulation of inflammatory cytokines and ROS/NO generation observed between septic patients and healthy volunteers were influenced by the expression of CD163 on monocytes. In general, CD163+ monocytes produced higher amounts of TNF-α and IL-6 and lower amounts of ROS and NO than did CD163− monocytes. An interaction between the expression of CD163 with cytokine production was found upon stimulation with LPS or bacteria, with CD163+ monocytes producing higher amounts of cytokines in both patients and healthy volunteers. An interaction between the expression of CD163 and ROS generation was also found after *S. aureus* and *P. aeruginosa* stimulation. In this case, differences between CD163+ and CD163− cells were only observed in septic patients; under both bacterial stimuli, ROS generation was higher in sepsis patients than in healthy volunteers for both CD163+ and CD163− monocytes.

Detection of higher levels of inflammatory cytokines in CD163+ cells than in CD163− cells was unexpected because of the anti-inflammatory role of alternatively activated macrophages [17]. However, this finding is consistent with the concept of a dual role of CD163+ monocytes in sepsis. CD163 may be important for controlling inflammation by removing free hemoglobin secondary to hemolysis and converting heme to its anti-inflammatory metabolites, but it also may function as a sensor of bacteria [30]. Accordingly, Fabriek and coworkers demonstrated the binding of Gram-positive and Gram-negative bacteria to CD163 and induction of inflammatory cytokines in CD163-expressing CHO cells and suppression of bacteria-induced cytokines in human monocytes by blocking antibodies against CD163 [19]. Supporting our results with septic patients, we observed that modulation of cytokines production in a model of LPS tolerance occurred regardless of the expression of CD163 on monocytes cell surface [31].

## Conclusions

We demonstrated that monocytes from septic patients, which have impaired inflammatory cytokine production, display potent phagocytic activity and increased ROS and NO generation. This modulation represents a state in which the host attempts to control the initial systemic inflammatory response while maintaining control over infection. As we previously suggested, this modulation may represent the return to homeostasis in cases of successful antimicrobial therapy and recovery of underlying disease. In contrast, failure to mount a robust inflammatory response may represent a state of immunosuppression in protracted patients [12].
